# Supplementary material for: Netcombin: An algorithm for constructing optimal phylogenetic network from rooted triplets
Source: PLoS One. 2020 Sep 18;15(9):e0227842. doi: 10.1371/journal.pone.0227842 (PMC7500971; doi:10.1371/journal.pone.0227842)
Supplement: S4 File — (ZIP) [file pone.0227842.s004.zip › SequenceData-Triplet/sequenceData-10-20-30-0-Triplet/length 200, numseq 40, rate 10^-7/3 seq.pdf]

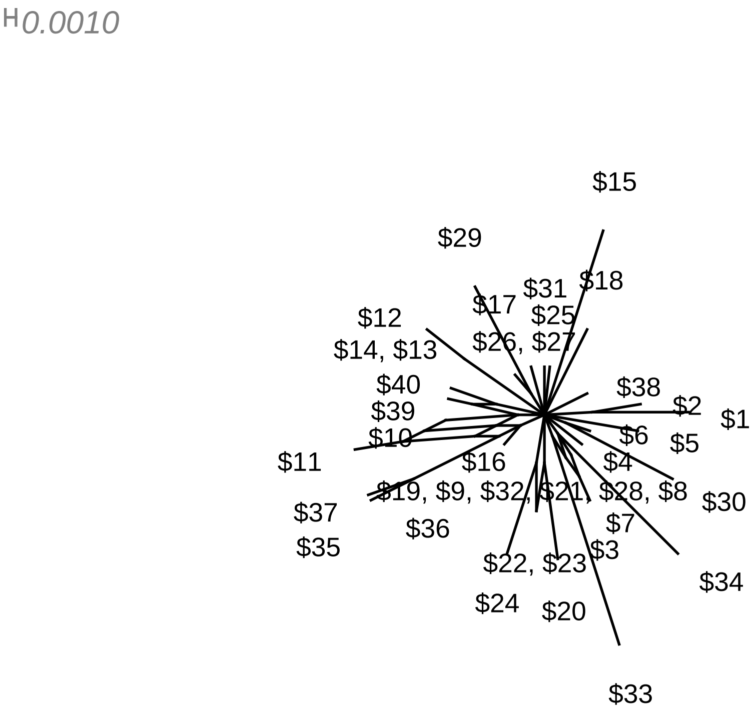
0.0010
$15
$29
$31
$18
$17
$25
$12
$26, $27
$14, $13
$40
$38
$2
$39
$1
$10
$6
$5
$11
$16
$4
$19, $9, $32, $21, $28, $8
$30
$37
$36
$7
$35
$3
$22, $23
$34
$24
$20
$33
